# A Challenging Diagnosis of Endometrial Stromal Sarcoma in a 50-Year-Old Patient: Case Report and Literature Review

**DOI:** 10.3390/diagnostics15243215

**Published:** 2025-12-16

**Authors:** Ana-Maria Haliciu, Cristina Furnică, Cristinel Ionel Stan, Raluca-Mihaela Gemanariu, Ioana Pavaleanu, Tudor Andrei Buțureanu, Andreea Pruteanu, Teodora Ana Balan, Bogdan Gabriel Anghel, Raluca Anca Balan

**Affiliations:** 1 Department of Morpho-Functional Sciences I, “Grigore T. Popa” University of Medicine and Pharmacy, 16 University Street, 700115 Iasi, Romania; anna_cefalan@yahoo.com (A.-M.H.); cristina.furnica@gmail.com (C.F.); cristi_stan00@yahoo.com (C.I.S.); balan.teodora-ana@d.umfiasi.ro (T.A.B.); anghelbogdan05@yahoo.com (B.G.A.); raluca.balan@umfiasi.ro (R.A.B.); 2 “Elena Doamna” Clinical Hospital of Obstetrics and Gynecology, 49 Elena Doamna Street, 700398 Iasi, Romania; ioana_pavaleanu@yahoo.com (I.P.); tudorandreib@gmail.com (T.A.B.); 3 Institute of Forensic Medicine, 4 Buna Vestire Street, 700455 Iasi, Romania; 4 Department of Mother and Child Medicine, “Grigore T. Popa” University of Medicine and Pharmacy, 16 University Street, 700115 Iasi, Romania; andreea.ioana.dragu@gmail.com

**Keywords:** low-grade endometrial stromal sarcoma, uterine sarcoma, hormonal therapy, hysterectomy, immunohistochemistry

## Abstract

**Background:** Low-grade endometrial stromal sarcoma (LG-ESS) is a rare uterine mesenchymal neoplasm characterized by indolent progression and strong hormonal responsiveness. Accurate diagnosis remains challenging due to its overlapping clinical, pathological, and imaging features with other benign or malignant uterine entities. **Case Presentation:** This article presents a case of LG-ESS in a 50-year-old woman, encompassing the clinical presentation, imaging features, histopathological and immunohistochemical findings, the surgical management and postoperative course, as well as a focused synthesis of the current literature on this pathology. The patient presented with recurrent abnormal uterine bleeding and secondary anemia. Imaging data, including magnetic resonance imaging (MRI), revealed a heterogeneous intracavitary lesion with cystic components, suggestive of submucosal fibroids. Surgical management via total hysterectomy with bilateral salpingo-oophorectomy was performed due to suspicion of sarcoma and the need to suppress hormonal stimulation. Histopathological and immunohistochemical evaluation confirmed LG-ESS. The patient had no lympho-vascular invasion, presenting a favorable postoperative evolution. **Conclusions:** This case highlights the need to maintain a strong clinical suspicion for uterine sarcomas in patients presenting with atypical bleeding and presumed fibroids, especially among perimenopausal women. A multidisciplinary approach, including imaging, surgery, pathology, molecular profiling, and oncology, is essential for accurate diagnosis and optimal management.

## 1. Introduction

Low-grade endometrial stromal sarcoma (LG-ESS) is a rare uterine malignancy, accounting for a small subset of mesenchymal tumors, composed of cells that resemble proliferative-phase endometrial stroma, exhibiting infiltrative growth or lymphovascular invasion [1,2]. LG-ESS typically progresses slowly and shows a high sensitivity to hormonal influences. Usually, LG-ESS involves uterine corpus and less the cervix [1]. Despite its relatively indolent behavior, LG-ESS can recur and metastasize, making accurate diagnosis and appropriate management essential [3,4,5,6]. This article presents a case of LG-ESS in a 50-year-old patient, alongside an overview of uterine sarcomas and a literature review, aiming to enhance clinical understanding of this uncommon neoplasm [6,7].

LG-ESS is the second most common uterine mesenchymal malignancy after leiomyosarcoma, typically affecting premenopausal and perimenopausal women, though it occurs at a wide age range, also being reported in younger individuals. Risk factors include obesity, diabetes, early menarche, prolonged tamoxifen use or unopposed estrogen therapy, and prior pelvic radiation [7,8,9,10,11].

Clinical presentation often involves abnormal uterine bleeding, particularly in postmenopausal or perimenopausal women, pelvic pain, dysmenorrhea, or, less commonly, mass-effect symptoms including urinary or bowel obstruction, although up to 25% of patients are asymptomatic at diagnosis. Extrauterine spread is observed in roughly one-third of cases, most commonly involving the lung, nodes, and ovaries, especially when associated with endometriosis [12,13,14,15,16,17].

The diagnosis of LG-ESS is often delayed due to its nonspecific clinical and radiological features. Unlike endometrial carcinomas, LG-ESS cannot be reliably diagnosed via hysteroscopy or endometrial curettage, as sampling is often insufficient and fails to capture the infiltrative margins [18,19,20]. Definitive diagnosis requires histopathological evaluation of the entire tumor and its interface with adjacent myometrium, as well as an immunohistochemica assessment, LG-ESS being positive for CD10 [21,22], IFITM1 [22], WT1, ER, PR, several specific keratins, smooth muscle markers, sex cord markers, as well as Beta catenin [23]. Imaging modalities such as ultrasound, computed tomography (CT), and magnetic resonance imaging (MRI) provide limited specificity [17,24,25,26,27,28,29].

This article presents an in-depth case of LG-ESS in a 50-year-old woman, encompassing the clinical presentation, diagnostic workup, imaging features, histopathological and immunohistochemical findings, as well as the surgical management and postoperative course. In addition, it provides a focused synthesis of the current literature regarding epidemiology, pathogenesis, molecular alterations, and imaging features of LG-ESS, along with an overview of therapeutic strategies and long-term follow-up recommendations. Comparative references to high-grade endometrial stromal sarcoma (HG-ESS) are integrated throughout, highlighting the distinct biological behavior, prognostic implications, and management considerations between the two entities. To the best of our knowledge, there is a limited number of cases with LG-ESS, published as case reports, case series or retrospective studies, over the last decades [14,21,25,30,31,32,33,34,35,36,37,38,39,40,41,42].

## 2. Case Presentation

A 50-year-old nonsmoking woman presented to the gynecology department with abnormal uterine bleeding, hypogastric pain, and clinical features suggestive of secondary anemia. Her medical history was notable for uterine fibroids diagnosed via ultrasound 10 years earlier, for which she had received multiple blood transfusions due to chronic menometrorrhagia and anemia. Additional history included inguinal hernioplasty and a previous left femur fracture. Obstetric history revealed seven pregnancies, resulting in four term vaginal deliveries and three abortions (one spontaneous, two elective). Menarche occurred at age 13, and due to recurrent abnormal bleeding, the date of her last menstrual period could not be ascertained.

### 2.1. Clinical Examination

On pelvic examination, the vaginal mucosa and cervix appeared unremarkable on gross inspection, with no palpable cervical lesions or abnormalities. In contrast, bimanual palpation revealed an enlarged, globular, and tender uterus.

### 2.2. Imaging Findings

**Figure 1:**
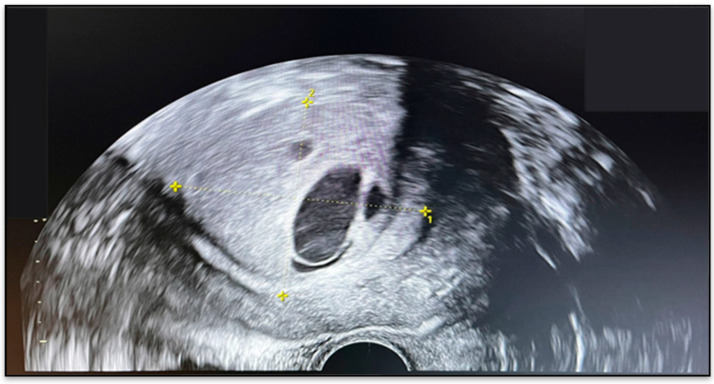
Transvaginal ultrasound showing an intracavitary uterine mass.

Ultrasound examination revealed a uterus measuring 103 × 78 mm, with heterogeneous echotexture and lobulated outer contours. An intracavitary mass with atypical sonographic features was identified, measuring 54 × 47 mm, suggestive of a leiomyoma (Figure 1). An additional anterior myometrial fibroid nodule measured 27 mm. The right ovary harbored a 29 mm cystic lesion, while the left ovary appeared unremarkable.

**Figure 2:**
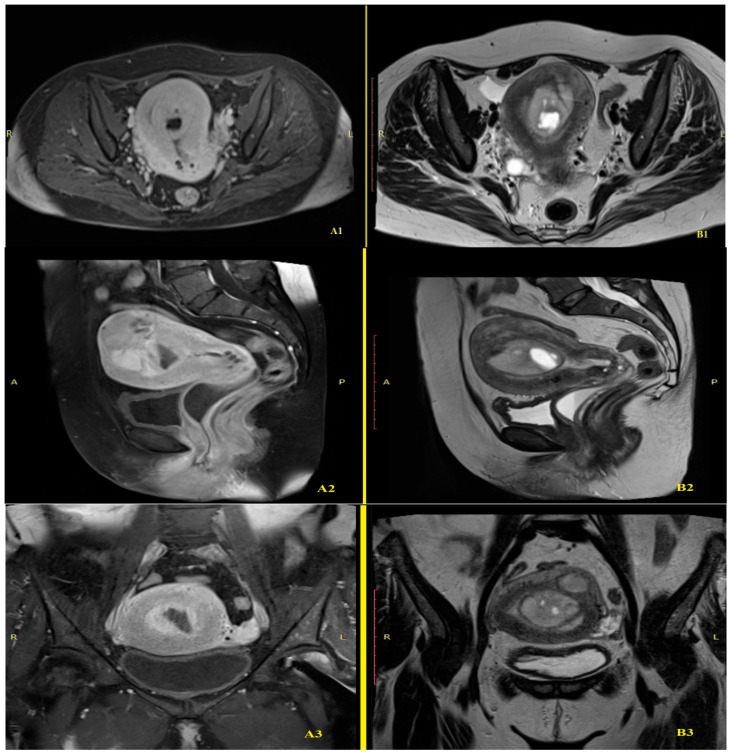
MRI comparison of T1-weighted post CIV: A, anterior; P, posterior; L, left; R, right; ((**A1**): transverse, (**A2**): sagittal, (**A3**): coronal) and T2-weighted ((**B1**): transverse, (**B2**): sagittal, (**B3**): coronal) sequences.

Pelvic contrast-enhanced MRI demonstrated a globally enlarged uterus (83 × 82 × 81 mm) with heterogeneous myometrial signal intensity, attributable to the presence of well-defined intramyometrial and submucosal nodules exhibiting higher signal intensity than the surrounding myometrium. Internal cystic areas were present in several of the lesions (Figure 2). The largest intracavitary nodule measured 58 × 35 mm. The endometrial stripe measured up to 10 mm in thickness, with intracavitary and vaginal fluid levels reaching 17 mm, interpreted as retained menstrual blood. Small Nabothian cysts, up to 7 mm in diameter, were observed in the cervix. Periuterine varices were more prominent on the left side. The right ovary contained two simple cysts measuring 20 mm and 18 mm, while the left ovary was homogeneous, measuring 17 mm. A thin layer of intraperitoneal fluid (15 mm) was present in the right lateral uterine region. No pelvic or inguinal lymphadenopathy was identified.

### 2.3. Initial Diagnosis and Management

The initial clinical diagnosis was hemorrhagic uterine leiomyoma associated with secondary anemia. In light of imaging findings suggestive of atypical fibroid degeneration and the possibility of an underlying uterine sarcoma, the patient underwent a total abdominal hysterectomy with bilateral salpingo-oophorectomy. Intraoperatively, the uterus appeared enlarged but without serosal nodules, surface irregularities, significant adhesions, or visible pelvic lymphadenopathy. The adnexa were grossly unremarkable, and no peritoneal or parametrial lesions suggestive of extrauterine spread were identified. The procedure served both definitive diagnostic and therapeutic purposes, while also aiming to eliminate potential hormonal stimulation in the context of a presumed estrogen-sensitive tumor. Sentinel lymph node (SLN) mapping was not performed because the preoperative clinical and imaging assessment suggested an atypical uterine mass with a working diagnosis of degenerating leiomyoma or a possible sarcoma. According to current ESGO and NCCN guidelines, SLN mapping is not recommended for suspected uterine sarcomas or fibroid-like tumors, as nodal assessment does not modify staging, treatment, or prognosis. Intraoperatively, no suspicious or enlarged lymph nodes were identified, further supporting the decision not to perform SLN mapping. Following the postoperative histopathological confirmation of low-grade endometrial stromal sarcoma (LG-ESS), re-evaluation of the case confirmed that lymph node assessment would still not have been indicated, as LG-ESS rarely metastasizes to lymph nodes and lymphadenectomy or SLN mapping does not improve clinical outcomes.

Fertility preservation was not considered, as the patient had completed childbearing and expressed no desire for future pregnancies. The postoperative course was uneventful, with gradual clinical improvement. Hemoglobin levels increased to 9.6 g/dL, and postoperative follow-up is ongoing to monitor for recurrence or metastatic spread.

#### 2.3.1. Pathological and Immunohistochemical Findings

**Figure 3:**
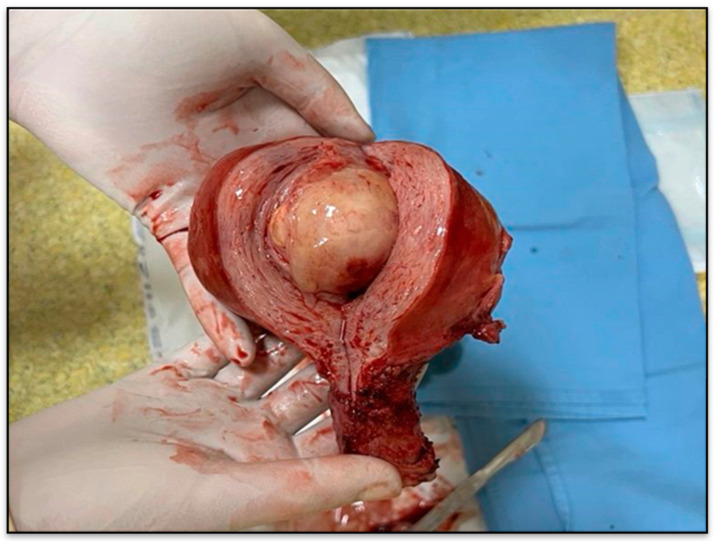
Gross specimen of the uterus with tumor nodule.

Gross description revealed a soft, gelatinous, yellow-tan to white nodular tumor measuring 75/55/48 mm, with heterogeneous appearance and areas of necrosis (Figure 3).

**Figure 4:**
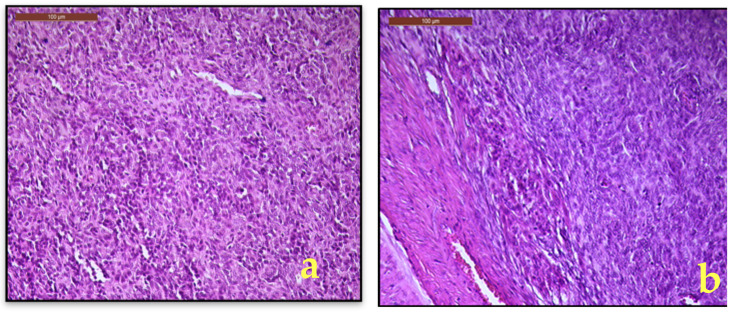
(**a**). LG-ESS, monotonous spindle to oval cells with minimal nuclearatypia; (**b**). Myometrial interface with diffuse and heterogeneous infiltration of the myometrium (H&E, 20×).

Microscopic examination revealed a mesenchymal proliferation composed of irregular islands of small, oval cells with a monotonous morphology, scant cytoplasm, with small round to spindle nuclei with minimal atypia (Figure 4a) and foci of sex cord-like growth. Some of the tumor cells are arranged around small arterioles, closely resembling proliferative-phase endometrial stroma. Foamy histiocytes and fibromyxoid differentiation are also seen. The tumor exhibited diffuse and heterogeneous infiltration of the myometrium (Figure 4b). In the right parametrium, only several cystically dilated glands are observed, without morphological aspects of lymphovascular invasion. Additional histopathological findings included focal adenomyosis, endocervical polyp, papillary endocervicitis, Nabothian cysts, and paratubal cysts.

The morphological description, along with the clinical and imaging aspects, were compatible with low grade endometrial stromal sarcoma, excluding endometrial stromal nodule, due to invasive growth pattern or other monomorphic spindle cell neoplasm.

**Figure 5:**
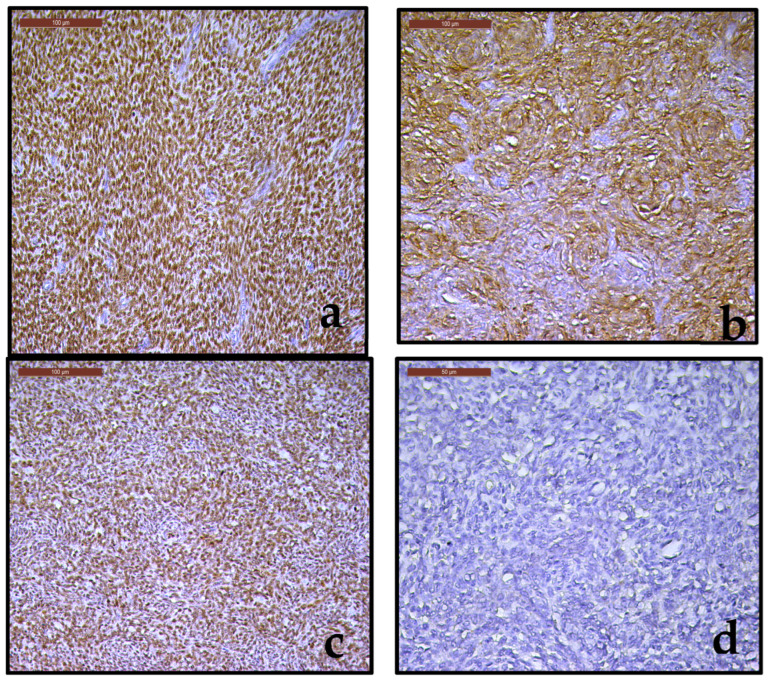
(**a**–**d**). (**a**). Strongly immunoreactive for WT1 (20×); (**b**). Strong immunoreactivity for CD10 (20×); (**c**). Intense and diffuse nuclear expression for p53 (20×); (**d**). Negative immunostaining for cyclin D1 (20×).

To confirm the histopathological diagnosis and to rule out a high grade endometrial stromal sarcoma, an immunohistochemical examination was performed, evaluating the following markers: WT1 (Figure 5a), CD10 (Figure 5b), over-expression pattern for p53 (intense and diffuse nuclear staining) in 80% of tumor cells (Figure 5c), absence of immunostaining for cyclin D1 (Figure 5d). The positive immunoexpression for WT1, CD10, the absence of immunoexpression for cyclin D1, aspects complemented by the specific pattern of p53 were consistent with the data reported in the literature and support our histopathological diagnosis of LG-ESS. Final histopathological diagnosis and pathological staging was low grade endometrial stromal sarcoma, pT1B Nx.

#### 2.3.2. Follow-Up

Following surgery and confirmation of the histopathological and immunohistochemical diagnosis, the patient was enrolled in a multidisciplinary follow-up program in accordance with established sarcoma and LG-ESS surveillance protocols. During the first two years, surveillance consisted of clinical assessment and pelvic examination every 6 months, complemented by transvaginal ultrasound, which is an accepted and widely used modality for routine pelvic follow-up in completely resected stage I LG-ESS. Chest imaging was performed at the same 6-month intervals.

Because the tumor was completely resected (FIGO stage I) with negative margins and no lymphovascular or nodal involvement, no adjuvant hormonal therapy or pelvic radiotherapy was initiated, consistent with current evidence restricting these treatments to recurrent or advanced disease. Throughout the first two years of surveillance, the patient remained asymptomatic, with no clinical or imaging signs of recurrence or metastasis. According to standard LG-ESS guidelines, follow-up will continue every 6 months until completing five years, followed by annual assessments thereafter, given the known potential for very late recurrences.

## 3. Discussion

Low-grade endometrial stromal sarcoma (LG-ESS) is an uncommon uterine malignancy, representing a small subset of mesenchymal tumors, being considered as the second most frequent type of uterine sarcoma [10]. It consists of cells resembling the endometrial stroma of the proliferative phase and is characterized by infiltrative growth or lymphovascular invasion [1,2]. LG-ESS generally has an indolent clinical course and demonstrates marked sensitivity to hormonal regulation. This article reports a case of LG-ESS in a 50-year-old woman, detailing the clinical presentation, imaging characteristics, histopathological and immunohistochemical findings, surgical treatment, and postoperative course, along with a concise review of the current literature on this condition. To the best of our knowledge, only a limited number of LG-ESS cases have been documented in the literature over the past few decades, primarily as case reports or small case series [32,43,44].

### 3.1. Pathologic Features

Uterine sarcomas are a rare and heterogeneous group of malignancies that originate from either the endometrial stroma or myometrial connective tissue components. Compared to the more prevalent endometrial carcinomas of epithelial origin, uterine sarcomas demonstrate more aggressive biological behavior and are typically associated with a poorer prognosis. Endometrial stromal tumors, a subset of uterine mesenchymal neoplasms, represent a histological spectrum ranging from benign stromal nodules to highly aggressive undifferentiated sarcomas [3].

Although ESS collectively account for less than 1% of all uterine malignancies, they represent 7–25% of uterine sarcomas, with an estimated annual incidence of 0.19–2 per 100,000 women, with a gradually increasing trend over the past decades. ESS typically affects premenopausal and perimenopausal women, with a mean age at diagnosis of 46 years (range 18–83), in contrast to the broader uterine sarcoma population, where the mean age at diagnosis is around 60 years [3,4,5,8,45].

According to the World Health Organization (WHO) classifications, the understanding of endometrial stromal tumors has evolved significantly over the past two decades. The 2003 WHO classification (historical terminology) categorized these tumors into three entities: benign endometrial stromal nodules (ESNs), low-grade endometrial stromal sarcoma (LG-ESS), and undifferentiated endometrial sarcoma (UES, historical term), based primarily on histologic features such as cytologic atypia and mitotic activity [3,4].

Historically, classification was heavily dependent on mitotic index. A mitotic count below 10 mitoses per 10 high-power fields (HPF) was associated with a favorable prognosis (up to 100% 5-year survival), while higher mitotic activity correlated with significantly poorer outcomes (approximately 55%). However, mitotic rate is now recognized as an unreliable prognostic indicator and is no longer a central diagnostic criterion [46].

Subsequent refinements occurred as molecular understanding expanded. Chang et al. [45] and Kurihara et al. [47] proposed subdivisions within undifferentiated sarcomas. A major diagnostic breakthrough occurred in 2012, with identification of the t (10;17) (q22;p13) YWHAE-FAM22A/B (NUTM2A/B) fusion, now considered the molecular hallmark of high-grade endometrial stromal sarcoma (HG-ESS). Consequently, the 2014 WHO classification revised the system into four distinct categories ESN, LG-ESS, HG-ESS, and undifferentiated uterine sarcoma (UUS) integrating histopathologic, immunohistochemical, and molecular features [5,6,7,8,11,45,47]. This update explicitly replaced the older term UES with UUS, reflecting a shift from morphology-based diagnosis to one informed by molecular signatures. HG-ESS was recognized as a distinct entity characterized by round-cell morphology and associated genetic alterations, particularly YWHAE-NUTM2 fusions, while LG-ESS and HG-ESS account for approximately 86% and 14% of endometrial stromal sarcomas, respectively [6,7,8].

In 2018, additional molecularly defined groups were described-especially tumors harboring BCOR internal tandem duplications or BCOR-related gene fusions. However, these did not constitute a separate WHO diagnostic category, but were incorporated into HG-ESS or UUS, depending on morphology and molecular profile [7,8,11]. Therefore, references to a “third group of ESS” represent literature-based molecular refinements, not an official WHO classification. The WHO 2020 classification further consolidated and refined these categories, emphasizing molecular alterations as essential diagnostic criteria:

- ESN: benign, non-invasive proliferation
- LG-ESS: typically associated with JAZF1, PHF1, MEAF6-PHF1, and related fusions; generally indolent
- HG-ESS: defined by YWHAE-NUTM2, BCOR, or ZC3H7B-BCOR fusions; aggressive clinical behavior
- UUS: lacks specific lineage differentiation or defining molecular markers; highly aggressive [1]

The evolution of WHO classifications demonstrates that mitotic index is no longer central, while molecular testing is now essential for accurate diagnosis, particularly for distinguishing LG-ESS from HG-ESS and from UUS. Macroscopically, LG-ESS appears as poorly defined, soft, yellow-tan to white nodules extending from the endometrium into the myometrium [9]. It may also present with submucosal or intramural growth, causing uterine cavity distortion, hemorrhage, necrosis, or cystic degeneration [9,10,11,12,13]. In some cases, LG-ESS may mimic a well-circumscribed mass; thus, extensive sampling of the tumor-myometrial interface is essential to exclude an endometrial stromal nodule (ESN) [48].

Histologically, LG-ESS often demonstrates submucosal or intramural growth with poorly defined margins and infiltrative projections into the myometrium and parametrial tissue, creating a distinctive worm-like pattern. The tumor consists of monotonous small oval to spindle-shaped cells arranged around spiral arteriole-like vessels, resembling proliferative endometrial stroma. The cells show minimal cellular atypia, vesicular nuclear chromatin, and sparse cytoplasm. The mitotic index is typically low (≤5 mitoses per 10 high-power fields), though higher counts can occur [9,10,11,12,13]. Collagen bands and foamy histiocytes may be interspersed within the lesion. Smooth muscle differentiation can be present, often displaying a characteristic starburst pattern with collagen bands radiating outward toward the periphery of the nodule. Additional reported patterns of differentiation include fibromyxoid, sex cord-like, epithelioid, rhabdoid, endometrioid glandular structures, pseudopapillary formations, clear cells, bizarre cells, and foci of adipose tissue [18]. The differential diagnosis of low-grade endometrial stromal sarcoma (LG-ESS) includes several histopathologic entities (Table 1) [1,37].

ESNs are benign tumors occurring most frequently in perimenopausal women, though they may arise across a wide age range. The most common presenting symptom is abnormal uterine bleeding, although up to 10% of cases are discovered incidentally during hysterectomy for unrelated conditions. ESNs can be intramural, submucosal, or polypoid and macroscopically appear as soft, yellow-tan, well-circumscribed but unencapsulated lesions. While focal hemorrhage, necrosis, or cystic changes may occasionally be present, myometrial and lymphovascular invasion must be entirely absent for a definitive diagnosis [11].

Microscopically, ESNs resemble proliferative-phase endometrial stroma, displaying minimal cytologic atypia and very low mitotic activity (typically ≤5 mitoses/10 HPF).

Immunohistochemically, ESNs show strong CD10 and vimentin positivity, and variably positive ER and PR staining. WT1 is usually absent or only focally expressed, in contrast to the diffuse WT1 positivity that characterizes LG-ESS. β-catenin demonstrates a membranous staining pattern in ESN, while nuclear accumulation is not expected and, when present, favors LG-ESS over ESN. Cytokeratin and SMA staining is typically focal or weak, and desmin is usually negative [11,12,49].

Recurrence is rare but has been documented, underscoring the need for thorough margin evaluation. A definitive diagnosis requires complete assessment of the entire nodule–myometrium interface in hysterectomy specimens [11,12,13].

High-grade endometrial stromal sarcomas (HG-ESS) frequently present with extrauterine spread and demonstrate an aggressive clinical course, with median progression-free survival of 7–11 months and overall survival of 11–23 months as reported by Lee et al. [50]. Garg et al. [51] likewise reported significantly poorer 5-year survival for HG-ESS compared with LG-ESS. Prognosis in HG-ESS is less dependent on stage compared with LG-ESS, although cervical involvement portends a worse outcome. Zhang et al. [52] identified CA125 level, tumor size, and associated endometriosis as independent prognostic factors.

Histologically, HG-ESS consists of large round-cell morphology arranged in nests or gland-like structures, with high mitotic activity (>10–30 mitoses/10 HPF), necrosis, and an infiltrative polypoid growth pattern. Diagnosis requires combined histopathologic evaluation and confirmatory immunohistochemical and molecular testing, particularly to identify YWHAE-NUTM2 and BCOR-altered subsets [3].

### 3.2. Immunohistochemical Findings

A key histological feature distinguishing LG-ESS from ESN is the presence of infiltrative growth, accompanied by an immunophenotype that, although overlapping, shows several characteristic patterns. Immunohistochemically, LG-ESS typically expresses CD10, WT1, ER, PR, vimentin, IFITM1, and variably smooth muscle markers such as SMA (focal). Androgen receptor (AR) may also be positive in approximately 45% of cases, but its expression is variable and not considered a defining feature. Cyclin D1 is negative or expressed in <10% of tumor nuclei, an important discriminator from high-grade ESS, which shows strong, diffuse nuclear positivity (>70%).

CD10 is frequently expressed in LG-ESS but is not fully specific, as focal expression can also occur in cellular leiomyoma and leiomyosarcoma. Therefore, evaluation with additional markers including h-caldesmon, desmin, smooth muscle myosin heavy chain, transgelin, and oxytocin receptor is important when smooth muscle differentiation is a concern [25,53]. Ac-cording to Hwang et al. [20], an ER (+)/PR (+)/CD10 (+)/GEM (−)/h-caldesmon (−)/transgelin (−) im-munoprofile reliably distinguishes LG-ESS from uterine leiomyosarcoma [18,19,20,45,46,47].

LG-ESS may also express β-catenin, usually with membranous staining; nuclear accumulation is uncommon and, when present, suggests CTNNB1-related biology typical of a subset of LG-ESS rather than ESN. IFITM1 is another useful marker, with reported sensitivity of ~83% and specificity of ~70% [21].

Focal staining with low-molecular-weight cytokeratins (CAM5.2, MNF116, CK8/18) may be seen, although cytokeratin expression is typically weak and patchy rather than diffuse. Smooth muscle markers (SMA, desmin, h-caldesmon) may be positive in areas showing smooth muscle differentiation, whereas sex-cord markers (inhibin, calretinin, CD99, Melan-A, WT1) may be focally expressed in areas with sex-cord-like differentiation.

Low-grade endometrial stromal sarcoma typically shows negative staining for DOG1, BCOR, and FOXL2, and p53 expression generally follows a wild-type (mosaic) pattern [1,11,12].

ESNs typically show strong and diffuse staining for CD10 and vimentin, with variable positivity for ER and PR. WT1 is usually negative or only focally positive, in contrast to the diffuse nuclear WT1 staining characteristic of LG-ESS. Cytokeratin and SMA may show only focal/weak staining, while desmin is typically negative. β-catenin demonstrates a membranous staining pattern in ESN, nuclear accumulation is not expected and, when present, favors a diagnosis of LG-ESS rather than ESN [11,12,49].

HG-ESS typically lack ER, PR, and CD10 expression but show strong and diffuse cyclin D1 positivity (>70% of tumor nuclei), which is a key diagnostic discriminator. DOG1 may assist in distinguishing HG-ESS from mimics such as GIST, while epithelial membrane antigen and cytokeratin are usually negative [11,49].

LG-ESS usually shows strong expression of ER and PR, diffuse CD10 and vimentin positivity, and may express β-catenin (membranous pattern). UUS tends to lack hormone receptor expression and may demonstrate EGFR positivity [3].

In our case, the tumor showed strong and diffuse immunoreactivity for WT1 and CD10, with absent cyclin D1 expression. Immunohistochemical evaluation of p53 was per-formed using the DO-7 monoclonal antibody clone, which represents the standard assay in our institution. The staining pattern was consistent with a wild-type (mosaic) profile, characterized by scattered weak-to-moderate nuclear positivity in a minority of tumor cells, without diffuse strong overexpression or complete absence of staining. This pattern does not suggest an aberrant (mutation-type) p53 expression. Molecular TP53 sequencing or NGS analysis was not per-formed; therefore, genomic confirmation of TP53 status is not available. Importantly, because aberrant p53 expression is exceptionally uncommon in LG-ESS, p53 immunostaining is not typically considered an independent prognostic factor for this tumor type. The definitive diagnosis of LG-ESS was established solely through histopathological assessment and a comprehensive immunohistochemical panel, without molecular testing. This approach was justified because the tumor morphology and IHC profile were characteristic of LG-ESS, and the main diagnostic uncertainties were at the clinical and imaging levels rather than the microscopic level. In addition, molecular testing (FISH/RT-PCR/NGS) remains limited in availability in our setting, associated with substantially higher costs and prolonged turnaround times. Therefore, NGS is not routinely performed for LG-ESS and is typically reserved for diagnostically challenging cases demonstrating clear morphologic and IHC overlap with high-grade ESS or other uterine sarcomas.

### 3.3. Molecular Features

Molecularly, LG-ESS is characterized by recurrent chromosomal translocations, most notably t (7;17) (p15;q21), resulting in JAZF1-SUZ12 gene fusion, a defining molecular signature (Table 2). This fusion is present in approximately 75% of ESNs, 45% of LG-ESSs, and less commonly (14%) in HG-ESSs [37,49,54]. Additional fusions include JAZF1-PHF1, EPC1-PHF1, MEAF6-PHF1, BRD8-PHF1, EPC2-PHF1, and rare events such as MBTD1-CXorf67 and JAZF1-BCORL1. Some of these rearrangements, particularly those involving PHF1, have been associated with histological variants exhibiting sex cord-like differentiation [37,54,55].

HG-ESS is most commonly defined by the balanced t (10;17) (q22;p13) translocation, producing the YWHAE-NUTM2A/B (formerly FAM22A/B) gene fusion (Table 1). This rearrangement is highly specific for HG-ESS and has not been found in other gynecologic malignancies such as leiomyosarcoma, carcinosarcoma, or adenosarcoma. Tumors harboring this fusion typically show high-grade round-cell morphology, aggressive behavior, strong cyclin D1 expression, and reduced hormone receptor expression, making this fusion both a diagnostic and prognostic marker [55]. These tumors are negative for the translocations seen in LG-ESS (JAZF1-SUZ12), helping in differential diagnosis. A variant involving ZC3H7B-BCOR fusion has also been described, morphologically resembling myxoid leiomyosarcoma, and it was identified an internal tandem duplications of the BCOR gene in a subset of HG-ESS/USS, especially those with diffuse cyclin D1 expression, suggesting a distinct molecular subtype [25,56].

**Table 2:** Comparative features of ESN vs. LG-ESS vs. HG-ESS; AR, androgen receptor; BSO, bilateral salpingo-oophorectomy; CD10, common acute lymphoblastic leukemia antigen; CD117, c-kit proto-oncogene; ER, estrogen receptor; ESN, endometrial stromal nodule; ESS, endometrial stromal sarcoma; HG-ESS, high-grade endometrial stromal sarcoma; HPF, high-power field; IFITM1, interferon-induced transmembrane protein 1; LG-ESS, low-grade endometrial stromal sarcoma; OS, overall survival; PR, progesterone receptor; SMA, smooth muscle actin; TH, total hysterectomy; WT1, wilms tumor protein 1; BCOR, BCL6 Corepressor; UUS, Undifferentiated Uterine Sarcoma.

Features	LG-ESS	HG-ESS	ESN
Typical Age of Onset	40–55 years (perimenopausal women) [3,4,5,8,45,57,58]	Any adult age, often younger than UUS [11,25,50,51,52,54,56]	Most often perimenopausal, but can occur at any age [11,12,13]
Growth behavior	Indolent, slow-growing [9,10,11,12,13]	Aggressive, rapid progression [9,24,49,50,51,53,56]	Benign [11,12,13]
Molecular hallmark	t (7;17) (p15;q21) → JAZF1-SUZ12 (~45%), JAZF1-PHF1, EPC1-PHF1, MEAF6-PHF1, BRD8-PHF1, EPC2-PHF1, rare MBTD1-CXorf67, JAZF1-BCORL1 [37,54,55]	t (10;17) (q22;p13) → YWHAE-NUTM2 fusion (NUTM2A/NUTM2B), ZC3H7B-BCOR fusion; BCOR internal tandem duplications (ITDs) [25,55,56]	t (7;17) (p15;q21) → JAZF1-SUZ12 fusion in ~75% [37,54]
Histology	Small, uniform spindle cells; tongue-like infiltration [9,10,11,12,13]	Large round cells in nests; high cellularity, frequent necrosis [49,50,51]	Proliferative-phase stromal cells, minimal atypia; well-circumscribed, unencapsulated; no myometrial/lymphovascular invasion [11,12,13]
Mitotic index	<5 mitoses/10 HPF [9,10,11,12,13]	>10–30 mitoses/10 HPF [49,50,51]	≤5 mitoses/10 HPF [11,12,13]
Cyclin D1 Expression	Usually negative, Cyclin D1 <10% nuclei [9,10,11,12,13]	Cyclin D1 strong diffuse nuclear positivity (>70%) nuclei positive [9,24,49,50,51,53,56]	Negative [11,12,13]
Immunohistochemistry	CD10+, ER+, PR+, WT1+, vimentin+, actins+, IFITM1+; may express SMA, β-catenin, pancytokeratins; CD117, BCOR negative (non-rearranged); no BCOR protein overexpression, normal/p53 wild-type pattern (non-aberrant) [17,18,19,45,46,47,52]	ER−/PR− or only focally positive; BCOR overexpression in BCOR rearranged tumors (ZC3H7B-BCOR fusion, BCOR ITD); not all HG-ESS are BCOR-positive, CD10 variable or focal; negative for smooth-muscle markers. [9,24,49,50,51,53,56]	CD10+, ER+, PR+, and absence of infiltration, WT1 variably positive/weak/focal; AR−, SMA−, desmin−; vimentin+; β-catenin membranous; cytokeratins focal positive/weak; IFITM1+ (weak/focal positive), BCOR negative (non-rearranged pattern) [11,12]
Margins and invasion	Myometrial and lymphovascular invasion common [9,10,11,12,13]	Extensive myometrial and extrauterine invasion [9,24,49,50,51,53,56]	No invasion (diagnosis requires absence of myometrial and lymphovascular invasion) [11,12,13]
Common Sites of Recurrence	Pelvis, lungs, abdomen (late recurrence) [9,10,11,12,13]	Often extrauterine at diagnosis (e.g., lung, liver) [9,24,49,50,51,53,56]	Rare [11,12,13]
Recurrence risk	10–20%, can recur decades later [9,10,11,12,13]	High, often early post-treatment [9,24,49,50,51,53,56]	Very low [11,12,13]
Prognosis	Favorable (5-year survival ~98% for stage I) [45,59,60]	Poor (5-year OS 25–55%) [9,24,49,50,51,53,56]	Excellent [11,12,13]
Symptoms	Abnormal uterine bleeding, pelvic pain [3,4,5,6,7,8]	Often presents with extrauterine disease at diagnosis (up to 40–70%), abnormal bleeding, extrauterine spread [49,50,51]	Often asymptomatic; abnormal uterine bleeding if present [11,12,13]
Growth pattern	Infiltrative with ‘worm-like’ extensions [9,10,11,12,13]	Polypoid, infiltrative with necrosis [49,50,51]	Intramural, submucosal, or polypoid; well-circumscribed [11,12,13]
Treatment	Total hysterectomy + BSO; Adjuvant endocrine therapy is generally reserved for recurrent or advanced disease, not for completely resected stage I tumors. Often presents with extrauterine disease at diagnosis (up to 40–70%) [9,10,11,12,13,45,57,58]	Surgery (hysterectomy + BSO) followed by chemotherapy (anthracycline-based regimens such as doxorubicin ± ifosfamide). Radiotherapy may be considered for local control in selected cases [9,24,49,50,51,53,56]	Hysterectomy; no adjuvant therapy needed [11,12,13]

### 3.4. Imaging and Clinical Presentation

The imaging characteristics of LG-ESS frequently overlap with those of various benign and malignant gynecologic conditions, making accurate differential diagnosis essential. Endometrial carcinoma, which more commonly affects postmenopausal women, may mimic LG-ESS due to similar patterns of endo-myometrial invasion; however, these tumors are generally smaller, show less contrast enhancement, and exhibit a different growth pattern. Leiomyosarcoma typically appears as a larger, more heterogeneous myometrial mass with necrosis and hemorrhage, demonstrates high vascularity with rapid contrast enhancement, originates within the myometrium, and is more aggressive in behavior than LG-ESS. Degenerated leiomyomas may resemble LG-ESS on imaging, but classic leiomyomas are well-circumscribed, hypoechoic, and display high ADC values on DWI, features that support a benign diagnosis. Adenomyosis is characterized by ill-defined myometrial thickening with scattered myometrial cysts and low T2 signal intensity on MRI; unlike LG-ESS, it lacks a distinct mass, infiltrative growth, and significant mass effect [24,25].

LG-ESS presents with non-specific imaging features. On ultrasound, it may appear as a mass with multiseptated cystic areas or focal cystic degeneration. Ludovisi et al. reported that among uterine sarcomas, LG-ESS showed the highest rate of preserved normal endometrium (91.7%), regular tumor borders (60.4%), and low vascularity on Doppler assessment (42.5%) [61].

MRI has the highest sensitivity, typically revealing a polypoid endometrial mass with low signal intensity on T1-weighted images and heterogeneous high signal intensity on T2-weighted images. Lymphovascular invasion may present as “worm-like” low T2 signal bands traversing the myometrium, corresponding to preserved myometrial fibers. LG-ESS tends to enhance more avidly than endometrial carcinoma and often shows irregular margins, nodular myometrial extension, and extensive lymphovascular spread. In some cases, it may mimic an intramural leiomyoma with cystic degeneration [61,62,63,64].

Diffusion-weighted imaging (DWI) frequently shows increased signal intensity with reduced apparent diffusion coefficient (ADC) values, supporting a diagnosis of malignancy [65]. Advanced or high-grade lesions may display more hemorrhage, necrosis, and infiltration into adjacent structures such as the fallopian tubes, ligaments, and ovaries [24,25].

Transvaginal ultrasound may show one of four patterns: diffuse myometrial thickening, a central cavitary mass, a mural lesion, or a polypoidal projection into the endometrial cavity. These masses typically appear hypoechoic and heterogeneous, often with smooth, nodular, or ill-defined margins. Color Doppler imaging may show central or peripheral vascularity with a low resistive index (RI), consistent with increased tumoral blood flow [57,64,65].

MRI remains the preferred modality for evaluating LG-ESS due to its superior soft-tissue resolution, better delineation of tumor margins, and greater ability to assess the depth of myometrial invasion and extrauterine spread. Compared to endometrial carcinoma, LG-ESS typically appears larger and demonstrates more extensive invasion and contrast enhancement [16].

### 3.5. Treatment and Adjuvant Management of LG-ESS

#### 3.5.1. Surgical Management

Total hysterectomy with bilateral salpingo-oophorectomy (BSO) remains the standard primary treatment for LG-ESS, given its hormone-sensitive nature. When serosal involvement is present, en bloc resection is advised, morcellation is contraindicated due to the risk of peritoneal dissemination and decreased progression-free survival. New techniques using isolation bags or transvaginal retrieval may mitigate this risk in minimally invasive surgery [17,26,27,29,66]. Advanced or metastatic cases may require cytoreductive surgery; however, a study by Leath et al. [26] reported no survival benefit, indicating the importance of individualized surgical decision-making [26].

The role of ovarian preservation remains controversial. Chu et al. [27] proposed that loss of ERβ expression may indicate higher malignant potential, and that estrogen replacement therapy could be harmful [27]. Nonetheless, some studies suggest that ovarian preservation does not impact overall survival (OS) significantly. Ovary-sparing surgery may be considered in young women with stage I disease, especially those desiring fertility [28]. Fertility-sparing surgery is an option in carefully selected patients with stage IA disease but was noticed a higher recurrence risk in stage IB patients. The decision to preserve ovaries should be individualized, considering the trade-off between recurrence risk and quality-of-life benefits in younger women. Estrogen replacement therapy following oophorectomy is not recommended due to the risk of stimulating residual tumor cells [26,27]. Ovarian conservation can be considered only in premenopausal women with FIGO stage I disease, particularly those who wish to preserve hormonal function or future fertility. It is not recommended in stage II–IV disease due to the increased risk of occult ovarian involvement and hormonally driven stimulation of residual tumor cells.

In premenopausal women under age 50 with early-stage disease, ovarian conservation has not been shown to negatively affect survival [59]. For selected women under 35 years old with small, early-stage tumors, ovary-sparing hysterectomy may be considered, but should be individualized based on tumor size and patient preference [67]. Reported recurrence rates of 25–75% in early-stage ESS derive from heterogeneous cohorts that include multiple endometrial stromal sarcoma subtypes and therefore should not be directly extrapolated to pure LG-ESS. Nevertheless, LG-ESS is known for its long-term risk of pelvic or abdominal recurrence, often many years after initial treatment. Surgical resection of isolated metastases, including pulmonary lesions, may provide benefit in carefully selected patients [29].

Lymph node metastasis occurs in 7–10% of cases, primarily within the pelvis. However, routine pelvic and para-aortic lymphadenectomy is not recommended, as studies, including those by Shah et al. [17] and Chan et al. [9], found no significant difference in 5-year survival between node-positive and node-negative cases (86% vs. 95%). LG-ESS typically spreads via transperitoneal or hematogenous routes. Bulky nodes may be resected during cytoreduction [9,17].

#### 3.5.2. Adjuvant Hormonal Therapy

Given the high expression of ER and PR in LG-ESS, hormonal therapy is a key adjunct in advanced or recurrent disease. Therapeutic options include progestins (megestrol acetate, medroxyprogesterone), aromatase inhibitors (letrozole, anastrozole, exemestane), and GnRH analogs [58,60]. Response rates up to 82% have been reported in patients with microscopic residual disease and durable remissions exceeding 10 years. Progestins like megestrol acetate and medroxyprogesterone, or aromatase inhibitors, are administered for residual or recurrent disease. Mifepristone monotherapy has demonstrated a 25% disease stabilization rate. Combination regimens (megestrol with tamoxifen) may be more effective, though further studies are needed. Hormonal therapy is also recommended in recurrent disease, but optimal regimen and duration remain undefined [38,41]. Response to hormonal therapy may be influenced by variable AR expression, which could partly explain cases of endocrine resistance despite ER/PR positivity. Gadducci et al. [60] recommend a 24-month course of progestin therapy, while others advocate for five years of aromatase inhibition [39,60]. Comparative studies showed prolonged recurrence-free survival with gestagens (306.2 months) and aromatase inhibitors (153.1 months), compared to no hormonal therapy (90.8 months), although side effects (depression, weight gain, fluid retention) may limit long-term use [68,69].

Hormonal therapy is typically not required in completely resected stage I/II cases with negative margins but can be considered as an alternative. In contrast, it is strongly recommended in advanced-stage or recurrent settings [70]. Letrozole has shown effectiveness in recurrent cases. Tamoxifen and estrogen therapy are contraindicated due to stimulatory effects on tumor growth [29]. In our healthcare system, all major classes of endocrine agents used in LG-ESS (progestins, aromatase inhibitors, and GnRH analogs), are accessible within standard oncologic practice. Progestins and GnRH analogs can be prescribed without restrictive reimbursement barriers, whereas aromatase inhibitors are reimbursed through national oncology programs following multidisciplinary tumor board approval. Therefore, endocrine therapy can be administered when clinically indicated, in accordance with international recommendations, although the use of combination or experimental regimens generally requires individualized justification.

#### 3.5.3. Adjuvant Chemotherapy

Routine chemotherapy is not recommended in LG-ESS due to limited efficacy. A National Cancer Database study found no survival benefit in patients receiving chemotherapy, which was administered in only 4.8% of 2.414 cases. By contrast, patients with HG-ESS showed improved outcomes when treated with multi-agent chemotherapy [71].

It may be considered in progressive, metastatic, or hormone-resistant disease, with agents such as ifosfamide and doxorubicin showing partial responses. In a phase II study, ifosfamide treatment resulted in a 33% overall response rate. Chemotherapy may also be indicated in UES, although no definitive benefit has been shown to date. Active agents include ifosfamide, doxorubicin, gemcitabine, docetaxel, liposomal doxorubicin, and paclitaxel [3].

#### 3.5.4. Radiotherapy

The role of adjuvant pelvic radiotherapy in LG-ESS remains uncertain. Evidence derives almost entirely from small, retrospective, and often heterogeneous cohorts, many of which include mixed uterine sarcoma histologies rather than LG-ESS alone. Some retrospective studies report an improvement in locoregional recurrence-free survival, but no demonstrable benefit in overall survival [69]. A phase III trial involving patients with uterine sarcomas including only 28 cases of ESS showed a reduction in local recurrence rates with postoperative radiotherapy, yet no improvement in progression-free or overall survival. Given the typically indolent natural history of LG-ESS and the limited LG-ESS-specific data, adjuvant radiotherapy should be reserved for carefully selected high-risk cases, with acknowledgment of potential long-term toxicities. In recurrent or advanced disease, radiotherapy may be considered for local control or palliation of symptoms such as pain, bleeding, or compression of adjacent organs [72]. The SARCGYN study found that polychemotherapy followed by radiotherapy improved 3-year disease free survival compared to radiotherapy alone. Active agents include doxorubicin, ifosfamide, cisplatin, and paclitaxel, but response rates are low (5–27%) in recurrent settings [5,73].

#### 3.5.5. Emerging Therapies and Follow-Up

Targeted therapies under investigation include WT1-directed immunotherapy, as WT1 is expressed in up to 93% of ESS cases. Tyrosine kinase inhibitors like imatinib mesylate have shown early promise due to PDGFR and c-abl expression in certain tumors. Recurrence rates in early-stage disease range from 25% to 75%, with median time to recurrence of 65 months in stage I and 9 months in advanced disease. Long-term follow-up is essential, and treatment of recurrence may involve surgery, hormone therapy, chemotherapy, or targeted agents, depending on disease pattern and prior therapies. Surgical resection, hormone therapy, chemotherapy, or targeted agents may be considered based on recurrence pattern, previous therapies, and patient comorbidities [72,74].

### 3.6. Prognostic Factors

The prognosis is strongly influenced by tumor stage: early-stage disease (FIGO I) is associated with excellent outcomes, with 5- and 10-year survival rates of 98% and 89%, respectively, whereas advanced stages (III–IV) show lower survival (66%) and significantly higher recurrence rates (up to 76%) [45,59,60]. Additional factors with unclear prognostic significance include tumor size, mitotic index, depth of invasion, and cellular atypia [10,75].

Negative resection margins remain the most consistent predictor of favorable prognosis. Patients with completely excised stage I tumors show superior survival compared to those with stage II disease (84% vs. 62% at 5 years; 77% vs. 49% at 10 years) [76].

In the study by Nasioudis et al. [42], ovarian preservation in premenopausal women with LG-ESS confined to the uterus was linked to a higher risk of recurrence, though it did not negatively impact overall survival. As such, ovary-sparing surgery may be cautiously considered in carefully selected patients following detailed counseling [42].

LG-ESS generally follows a slow-growing, indolent course but carries a significant risk of late recurrence, often developing more than a decade after initial treatment, typically involving the pelvis, lungs, abdomen, or vagina [3,59].

Lymph node involvement, though infrequent, is associated with markedly poorer outcomes. One large cohort study reported a survival rate of 35.3% in node-positive patients versus 80.1% in those without nodal metastasis [9]. While the therapeutic benefit of lymphadenectomy remains unproven, identification of nodal involvement may have prognostic implications. In terms of hormonal influence, estrogen is known to promote tumor growth. Thus, oophorectomy is typically recommended, although recent data indicate that ovarian preservation may not compromise survival in young women with stage I disease [9,10,59,67,70,74,76].

#### Recurrence and Metastatic Spread

LG-ESS is characterized by an indolent course but a notable risk of recurrence, reported in 10–30% of cases, even decades after initial treatment. Recurrences typically involve the pelvis, lungs, or intra-abdominal sites, with approximately 60% presenting as distant or intra-abdominal metastases. Early-stage disease (FIGO I–II) is associated with longer disease-free survival (5.4–9.3 years), while advanced stages recur earlier (9 months) [40,77].

Recurrence may occur even in node-negative patients, and cytoreductive surgery remains the most effective therapeutic option in selected cases. Radiotherapy can be used for local control or palliation but does not prevent progression in most cases [78]. Hormonal therapy, using progestins or aromatase inhibitors, is the preferred strategy in residual, recurrent, or metastatic disease due to strong steroid receptor expression [79].

Emerging targeted therapies, such as tyrosine kinase inhibitors (imatinib), are under investigation. EGFR expression, present in up to 70% of ESS cases, suggests a potential therapeutic target [80]. HIPEC (hyperthermic intraperitoneal chemotherapy) is also being explored as a future approach in select recurrent settings [41].

## 4. Conclusions

LG-ESS is a rare, hormonally responsive uterine malignancy with indolent evolution but potential for delayed recurrence. This article presents a documented case of LG-ESS in a 50-year-old patient, emphasizing key clinical, histopathological, and imaging features that supported diagnosis and management. Through this case and a focused literature synthesis, we explore the diagnostic challenges posed by LG-ESS, its typical immunohistochemical and molecular profile, and the current standards of treatment, primarily surgical resection, with selective use of hormonal therapy. Highlighting both the individualized approach to care and the need for long-term follow-up, this report contributes to the growing understanding of optimal strategies for managing LG-ESS. This report contributes to the growing body of literature supporting early recognition and tailored therapy for LG-ESS, with attention to fertility considerations, recurrence risk, and the evolving role of hormone therapy.

## Figures and Tables

**Table 1:** Differential diagnosis of LG-ESS; ESN, endometrial stromal nodule; HG-ESS, high-grade endometrial stromal sarcoma; UTROSCT, uterine tumor resembling ovarian sex cord tumor; IHC, immunohistochemistry.

ESN
Absence of myometrial invasion, or no more than 3 tongues of invasion, each <3 mm No lymphovascular invasion Histology shows well-circumscribed, non-infiltrative nodules resembling proliferative endometrial stroma. IHC profile: CD10+, ER+, PR+, vimentin+ (variable intensity); WT1: usually focal or negative (important distinction from LG-ESS); Cytokeratin, SMA: focal/weak; Desmin: negative; β-catenin membranous, not nuclear (nuclear → favors LG-ESS with CTNNB1 mutation)
HG-ESS
Significant cytologic atypia High mitotic activity (>10–30 mitoses/10 HPF) Frequent necrosis Immunophenotype: Cyclin D1: strong, diffuse nuclear positivity (>70% of tumor nuclei); BCOR: strong, diffuse nuclear positivity in BCOR-rearranged cases; ER/PR: typically negative; CD10: usually negative or only focal Molecular alterations: YWHAE–NUTM2A/B fusion (hallmark); ZC3H7B–BCOR fusion; BCOR internal tandem duplications (ITDs); HG-ESS categories correspond to these genetic events.
CELLULAR LEIOMYOMA
Uniform smooth muscle cells with fascicular architecture Thick-walled blood vessels Cleft-like spaces No infiltrative margins Molecular profile: Lacks endometrial stromal fusions (JAZF1, PHF1) IHC: SMA+, desmin+, h-caldesmon+; CD10: may be focal but not diffuse; ER/PR: can be positive
LEIOMYOSARCOMA
Marked cytologic atypia High mitotic index Coagulative tumor cell necrosis Thick-walled, hyalinized vessels Molecular profile: Does NOT harbor JAZF1 or PHF1 fusions IHC: SMA+, desmin+, h-caldesmon+ (strong, diffuse); CD10: usually negative; ER/PR: variable
UTROSCT
Lacks conventional endometrial stromal differentiation Architectural patterns mimicking ovarian sex cord tumors Molecular alterations: Gene fusions involving ESR1 or GREB1 (e.g., ESR1-NCOA2, GREB1-NCOA2) IHC: Positive for sex cord markers: inhibin, calretinin, SF-1, CD99; CD10: usually negative; ER/PR variably positive
ENDOMETRIAL POLYP
Does not show expansile stromal growth Does not displace surrounding endometrium No infiltrative pattern Often contains thick-walled vessels and fibrous stroma
GLAND-POOR ADENOMYOSIS
No confluent stromal proliferation Does not displace or infiltrate myometrium Endometrial stroma present only around ectopic glands

## Data Availability

The original contributions presented in this study are included in the article. Further inquiries can be directed to the corresponding author.

## Abbreviations

The following abbreviations are used in this manuscript:

ADC	Apparent diffusion coefficient;
AR	Androgen receptor;
BCOR	Bcl6 corepressor;
BSO	Bilateral salpingo-oophorectomy;
CA125	Cancer antigen 125;
CD10	Common acute lymphoblastic leukemia antigen;
CD117	C-kit proto-oncogene;
CT	Computed tomography;
DWI	Diffusion-weighted imaging;
EGFR	Epidermal growth factor receptor expression;
ER	Estrogen receptor;
ESN	Endometrial stromal nodule;
ESS	Endometrial stromal sarcoma;
HG-ESS	High-grade endometrial stromal sarcoma;
HIPEC	Hyperthermic intraperitoneal chemotherapy;
HPF	High-power field;
IFITM1	Interferon-induced transmembrane protein 1;
LG-ESS	Low-grade endometrial stromal sarcoma;
MRI	Magnetic resonance imaging;
OS	Overall survival;
PDGFR	Platelet-derived growth factor receptor;
PR	Progesterone receptor;
SLN	Sentinel lymph node;
SMA	Smooth muscle actin;
TH	Total hysterectomy;
UES	Undifferentiated endometrial sarcoma;
UUS	Undifferentiated uterine sarcoma;
WHO	World Health Organization;
WT1	Wilms tumor protein 1.

## References

[1] Kim K.-R., Lax S.F., Lazar A.J., Lonagcre T.A., Malpica A., Matias-Guiu X., Nucci M.R., Oliva E. (2020). Tumours of the uterine corpus. Female Genital Tumours. WHO Classification of Tumours.

[2] Masand R.P., Euscher E., Deavers M.T., Malpica A. (2013). Endometrial stromal sarcoma: A clinic-pathologic study of 63 cases. Am. J. Surg. Pathol..

[3] Rauh-Hain J.A., del Carmen M.G. (2013). Endometrial stromal sarcoma: A systematic review. Obs. Gynecol..

[4] Hanby A.M., Walker C., Tavassoli F.A., Devilee P. (2004). Pathology and Genetics: Tumours of the Breast and Female Genital Organs.

[5] Denschlag D., Thiel F.C., Ackermann S., Harter P., Juhasz-Boess I., Mallmann P., Strauss H.G., Ulrich U., Horn L.C., Schmidt D. (2015). Sarcoma of the Uterus. Guideline of the DGGG (S2k-Level, AWMF Registry No. 015/074, August 2015). Geburtshilfe Frauenheilkd.

[6] Lee C.H., Ou W.B., Mariño-Enriquez A., Zhu M., Mayeda M., Wang Y., Guo X., Brunner A.L., Amant F., French C.A. (2012). 14-3-3 fusion oncogenes in high-grade endometrial stromal sarcoma. Proc. Natl. Acad. Sci. USA.

[7] Ferreira J., Félix A., Lennerz J.K., Oliva E. (2018). Recent advances in the histological and molecular classification of endometrial stromal neoplasms. Virchows Arch..

[8] Conklin C.M.J., Longacre T.A. (2014). Endometrial stromal tumors: The new WHO classification. Adv. Anat. Pathol..

[9] Chan J.K., Kawar N.M., Shin J.Y., Osann K., Chen L.M., Powell C.B., Kapp D.S. (2008). Endometrial stromal sarcoma: A population-based analysis. Br. J. Cancer.

[10] Abeler V.M., Røyne O., Thoresen S., Danielsen H.E., Nesland J.M., Kristensen G.B. (2009). Uterine sarcomas in Norway. A histopathological and prognostic survey of a total population from 1970 to 2000 including 419 patients. Histopathology.

[11] Ali R.H., Rouzbahman M. (2015). Endometrial stromal tumours revisited: An update based on the 2014 WHO classification. J. Clin. Pathol..

[12] Leary A.F., Quinn M., Fujiwara K., Coleman R.L., Kohn E., Sugiyama T., Glasspool R., Ray-Coquard I., Colombo N., Bacon M. (2017). Fifth Ovarian Cancer Consensus Conference of the Gynecologic Cancer InterGroup (GCIG): Clinical trial design for rare ovarian tumours. Ann. Oncol..

[13] Usta T.A., Sonmez S.E., Oztarhan A., Karacan T. (2014). Endometrial stromal sarcoma in the abdominal wall arising from scar endometriosis. J. Obs. Gynaecol..

[14] Laufer J., Scasso S., Kim B., Shahi M., Mariani A. (2023). Fertility-sparing management of low-grade endometrial stromal sarcoma. Int. J. Gynecol. Cancer.

[15] Puliyath G., Nair M.K. (2012). Endometrial stromal sarcoma: A review of the literature. Indian J. Med. Paediatr. Oncol..

[16] Shah S.H., Jagannathan J.P., Krajewski K., O’Regan K.N., George S., Ramaiya N.H. (2012). Uterine sarcomas: Then and now. AJR Am. J. Roentgenol..

[17] Shah J.P., Bryant C.S., Kumar S., Ali-Fehmi R., Malone J.M., Morris R.T. (2008). Lymphadenectomy and ovarian preservation in low-grade endometrial stromal sarcoma. Obs. Gynecol..

[18] Nucci M.R. (2016). Practical issues related to uterine pathology: Endometrial stromal tumors. Mod. Pathol..

[19] Kontomanolis E.N., Sapantzoglou I., Nikolettos K., Kontogeorgi E., Lampraki V., Papageorgiou D., Perros P., Fasoulakis Z., Koulakmanidis A.-M., Daskalaki M.-A. (2025). Clinicopathological Predictors of Recurrence in Uterine Sarcomas-A Narrative Review. J. Clin. Med..

[20] Hwang H., Matsuo K., Duncan K., Pakzamir E., Pham H.Q., Correa A., Fedenko A., Mhawech-Fauceglia P. (2015). Immunohistochemical panel to differentiate endometrial stromal sarcoma, uterine leiomyosarcoma and leiomyoma: Something old and something new. J. Clin. Pathol..

[21] Ichimura T., Kasai M., Imai K., Yamauchi M., Fukuda T., Yasui T., Sumi T. (2022). A difficult to diagnose case of low-grade endometrial stromal sarcoma with smooth muscle differentiation treated with laparoscopic surgery: A case report. Mol. Clin. Oncol..

[22] Busca A., Gulavita P., Parra-Herran C., Islam S. (2018). IFITM1 Outperforms CD10 in differentiating low-grade endometrial stromal sarcomas from smooth muscle neoplasms of the uterus. Int. J. Gynecol. Pathol..

[23] Jung C.K., Jung J.H., Lee A., Lee Y.-S., Choi Y.-J., Yoon S.-K., Lee K.-Y. (2008). Diagnostic use of nuclear *β*-catenin expression for the assessment of endometrial stromal tumors. Mod. Pathol..

[24] Adiga C.P., Gyanchandani M., Goolahally L.N., Itagi R.M., Kalenahalli K.V. (2016). Endometrial stromal sarcoma: An aggressive uterine malignancy. J. Radiol. Case Rep..

[25] Rha S.E., Byun J.Y., Jung S.E., Lee S.L., Cho S.M., Hwang S.S., Lee H.G., Namkoong S.E., Lee J.M. (2003). CT and MRI of uterine sarcomas and their mimickers. AJR Am. J. Roentgenol..

[26] Leath C.A., Huh W.K., Hyde J., Cohn D.E., Resnick K.E., Taylor N.P., Powell M.A., Mutch D.G., Bradley W.H., Geller M.A. (2007). A multi-institutional review of outcomes of endometrial stromal sarcoma. Gynecol. Oncol..

[27] Gothwal M., Yadav G., Rao M., Singh P., Nalwa A. (2018). Low-Grade Endometrial Stromal Sarcoma in a Postmenopausal Woman with Third-Degree Uterovaginal Prolapse: A Rare Case with Review of the Literature. J. Midlife Health.

[28] Cui R., Yuan F., Wang Y., Li X., Zhang Z., Bai H. (2017). Clinicopathological characteristics and treatment strategies for patients with low-grade endometrial stromal sarcoma. Medicine.

[29] Amant F., Coosemans A., Debiec-Rychter M., Timmerman D., Vergote I. (2009). Clinical management of uterine sarcomas. Lancet Oncol..

[30] Ayhan A., Toptas T., Oz M., Vardar M.A., Kayikcioglu F., Ozgul N., Gokcu M., Simsek T., Tunc M., Meydanli M.M. (2021). Low-grade endometrial stromal sarcoma: A Turkish uterine sarcoma group study analyzing prognostic factors and disease outcomes. Gynecol. Oncol..

[31] Singhal S., Jayraj A.S., Dhamija E., Khurana S. (2023). Low-grade extrauterine endometrial stromal sarcoma arising from vaginal endometriosis: A case report and literature review. Korean J. Clin. Oncol..

[32] Stefanko D.P., Eskander R., Aisagbonhi O. (2020). Disseminated endometriosis and low- grade endometrioid stromal sarcoma in a patient with a history of uterine morcellation for adenomyosis. Case Rep. Obs. Gynecol..

[33] McCarthy A.J., Clarke B.A., McGilvray I., Dickson B.C., Khalili K., Chetty R. (2019). Metastatic low-grade endometrial stromal sarcoma of uterus presenting as a primary pancreatic tumor: Case presentation and literature review. Diagn. Pathol..

[34] Smith E.S., Jansen C., Miller K.M., Chiang S., Alektiar K.M., Hensley M.L., Mueller J.J., Abu-Rustum N.R., Leitao M.M. (2022). Primary characteristics and outcomes of newly diagnosed low-grade endometrial stromal sarcoma. Int. J. Gynecol. Cancer.

[35] Park J.Y., Kim D.Y., Kim J.H., Kim Y.M., Kim Y.T., Nam J.H. (2011). The impact of tumor morcellation during surgery on the outcomes of patients with apparently early low- grade endometrial stromal sarcoma of the uterus. Ann. Surg. Oncol..

[36] Nakabayashi A., Odaira K., Horibe Y., Kanno T., Akizawa Y., Tabata T. (2020). A case of unsuspected low-grade endometrial stromal sarcoma successfully treated with two minimally invasive surgeries. Gynecol. Minim. Invasive Ther..

[37] Makise N., Sekimizu M., Kobayashi E., Yoshida H., Fukayama M., Kato T., Kawai A., Ichikawa H., Yoshida A. (2019). Low-grade endometrial stromal sarcoma with a novel MEAF6-SUZ12 fusion. Virchows Arch..

[38] Ryu H., Choi Y.S., Song I.C., Yun H.J., Jo D.Y., Kim S., Lee H.J. (2015). Long-term treatment of residual or recurrent low-grade endometrial stromal sarcoma with aromatase inhibitors: A report of two cases and a review of the literature. Oncol. Lett..

[39] Dahhan T., Fons G., Buist M.R., Ten Kate F.J.W., van der Velden J. (2009). The efficacy of hormonal treatment for residual or recurrent low-grade endometrial stromal sarcoma. A retrospective study. Eur. J. Obs. Gynecol. Reprod. Biol..

[40] Bai H., Yang J., Cao D., Huang H., Xiang Y., Wu M., Cui Q., Chen J., Lang J., Shen K. (2014). Ovary and uterus-sparing procedures for low-grade endometrial stromal sarcoma: A retrospective study of 153 cases. Gynecol. Oncol..

[41] Comert G.K., Turkmen O., Kar I., Yucel O., Kilic C., Boran N., Basaran D., Karalok A., Turan T. (2019). Hormone therapy following surgery in low-grade endometrial stromal sarcoma: Is it related to a decrease in recurrence rate?. J. Chin. Med. Assoc..

[42] Nasioudis D., Ko E.M., Kolovos G., Vagios S., Kalliouris D., Giuntoli R.L. (2019). Ovarian preservation for low-grade endometrial stromal sarcoma: A systematic review of the literature and meta-analysis. Int. J. Gynecol. Cancer.

[43] Clair K., Wolford J., Veran-Taguibao S., Kim G., Eskander R.N. (2017). Primary low-grade endometrial stromal sarcoma of the omentum. Gynecol. Oncol. Rep..

[44] Niu S. (2022). Diagnostic challenges and considerations of low-grade endometrial stromal sarcoma (LGESS) outside the female genital tract. Transl. Cancer Res..

[45] Chang K.L., Crabtree G.S., Lim-Tan S.K., Kempson R.L., Hendrickson M.R. (1990). Primary uterine endometrial stromal neoplasms. A clinicopathologic study of 117 cases. Am. J. Surg. Pathol..

[46] Günter K., Matthias E. (2009). Uterine Sarkome und Mischtumoren: Handbuch und Bildatlas zur Diagnostik und Therapie.

[47] Kurihara S., Oda Y., Ohishi Y., Iwasa A., Takahira T., Kaneki E., Kobayashi H., Wake N., Tsuneyoshi M. (2008). Endometrial stromal sarcomas and related high-grade sarcomas: Immunohistochemical and molecular genetic study of 31 cases. Am. J. Surg. Pathol..

[48] Rabban J.T., Gilks C.B., Malpica A., Matias-Guiu X., Mittal K., Mutter G.L., Oliva E., Parkash V., Ronnett B.M., Staats P. (2019). Issues in the Differential Diagnosis of Uterine Low-grade Endometrioid Carcinoma, Including Mixed Endometrial Carcinomas: Recommendations from the International Society of Gynecological Pathologists. Int. J. Gynecol. Pathol..

[49] Mariño-Enriquez A., Lauria A., Przybyl J., Ng T.L., Kowalewska M., Debiec-Rychter M., Ganesan R., Sumathi V., George S., McCluggage W.G. (2018). BCOR Internal Tandem Duplication in High-grade Uterine Sarcomas. Am. J. Surg. Pathol..

[50] Lee C.H., Mariño-Enriquez A., Ou W., Zhu M., Ali R.H., Chiang S., Amant F., Gilks C.B., van de Rijn M., Oliva E. (2012). The clinicopathologic features of YWHAE-FAM22 endometrial stromal sarcomas: A histologically high-grade and clinically aggressive tumor. Am. J. Surg. Pathol..

[51] Garg G., Shah J.P., Toy E.P., Bryant C.S., Kumar S., Morris R.T. (2010). Stage IA vs. IB endometrial stromal sarcoma: Does the new staging system predict survival?. Gynecol. Oncol..

[52] Zhang Y.Y., Li Y., Qin M., Cai Y., Jin Y., Pan L.Y. (2019). High-grade endometrial stromal sarcoma: A retrospective study of factors influencing prognosis. Cancer Manag. Res..

[53] Park J.Y., Baek M.H., Park Y., Kim Y.T., Nam J.H. (2018). Investigation of hormone receptor expression and its prognostic value in endometrial stromal sarcoma. Virchows Arch..

[54] Tsuyoshi H., Yoshida Y. (2018). Molecular biomarkers for uterine leiomyosarcoma and endometrial stromal sarcoma. Cancer Sci..

[55] Micci F., Heim S., Panagopoulos I. (2021). Molecular pathogenesis and prognostication of “low-grade’’ and “high-grade” endometrial stromal sarcoma. Genes. Chromosomes Cancer.

[56] Kim Y., Kim D., Sung W.J., Hong J. (2022). High-Grade Endometrial Stromal Sarcoma: Molecular Alterations and Potential Immunotherapeutic Strategies. Front. Immunol..

[57] Nightingale K., Clough E., Goldsmith P., Burke J.R. (2024). Peritoneal inclusion cyst presenting as an umbilical hernia: Case report and systematic review of the literature. J. Surg. Case Rep..

[58] Reich O., Singer C., Hudelist G., Regauer S. (2007). Estrogen sulfotransferase expression in endometrial stromal sarcomas: An immunohistochemical study. Pathol. Res. Pract..

[59] Gadducci A. (2011). Prognostic factors in uterine sarcoma. Best Pract. Res. Clin. Obstet. Gynaecol..

[60] Gadducci A., Cosio S., Romanini A., Genazzani A.R. (2008). The management of patients with uterine sarcoma: A debated clinical challenge. Crit. Rev. Oncol. Hematol..

[61] Ludovisi M., Moro F., Pasciuto T., Di Noi S., Giunchi S., Savelli L., Pascual M.A., Sladkevicius P., Alcazar J.L., Franchi D. (2019). Imaging in gynecological disease (15): Clinical and ultrasound characteristics of uterine sarcoma. Ultrasound Obs. Gynecol..

[62] Santos P., Cunha T.M. (2015). Uterine sarcomas: Clinical presentation and MRI features. Diagn. Interv. Radiol..

[63] Sala E., Rockall A.G., Freeman S.J., Mitchell D.G., Reinhold C. (2013). The added role of MR imaging in treatment stratification of patients with gynecologic malignancies: What the radiologist needs to know. Radiology.

[64] Concin N., Matias-Guiu X., Vergote I., Cibula D., Mirza M.R., Marnitz S., Ledermann J., Bosse T., Chargari C., Fagotti A. (2021). ESGO/ESTRO/ESP guidelines for the management of patients with endometrial carcinoma. Int. J. Gynecol. Cancer.

[65] Huang Y.L., Ueng S.H., Chen K., Huang Y.T., Lu H.Y., Ng K.K., Chang T.C., Lai C.H., Lin G. (2019). Utility of diffusion-weighted and contrast-enhanced magnetic resonance imaging in diagnosing and differentiating between high- and low-grade uterine endometrial stromal sarcoma. Cancer Imaging.

[66] Ghirardi V., Bizzarri N., Guida F., Vascone C., Costantini B., Scambia G., Fagotti A. (2019). Role of surgery in gynaecological sarcomas. Oncotarget.

[67] Amant F., De Knijf A., Van Calster B., Leunen K., Neven P., Berteloot P., Vergote I., Van Huffel S., Moerman P. (2007). Clinical study investigating the role of lymphadenectomy, surgical castration and adjuvant hormonal treatment in endometrial stromal sarcoma. Br. J. Cancer.

[68] Deshmukh U., Black J., Perez-Irizarry J., Passarelli R., Levy K., Rostkowski A., Hui P., Rutherford T.J., Santin A.D., Azodi M. (2019). Adjuvant Hormonal Therapy for Low-Grade Endometrial Stromal Sarcoma. Reprod. Sci..

[69] Sampath S., Schultheiss T.E., Ryu J.K., Wong J.Y.C. (2010). The role of adjuvant radiation in uterine sarcomas. Int. J. Radiat. Oncol. Biol. Phys..

[70] Yamazaki H., Todo Y., Mitsube K., Hareyama H., Shimada C., Kato H., Yamashiro K. (2015). Long-term survival of patients with recurrent endometrial stromal sarcoma: A multicenter, observational study. J. Gynecol. Oncol..

[71] Seagle B.L.L., Sobecki-Rausch J., Strohl A.E., Shilpi A., Grace A., Shahabi S. (2017). Prognosis and treatment of uterine leiomyosarcoma: A National Cancer Database study. Gynecol. Oncol..

[72] Reed N.S., Mangioni C., Malmström H., Scarfone G., Poveda A., Pecorelli S., Tateo S., Franchi M., Jobsen J.J., Coens C. (2008). Phase III randomised study to evaluate the role of adjuvant pelvic radiotherapy in the treatment of uterine sarcomas stages I and II: An European Organisation for Research and Treatment of Cancer Gynaecological Cancer Group Study (protocol 55874). Eur. J. Cancer.

[73] Hoang L., Chiang S., Lee C.H. (2018). Endometrial stromal sarcomas and related neoplasms: New developments and diagnostic considerations. Pathology.

[74] Cheng X., Yang G., Schmeler K.M., Coleman R.L., Tu X., Liu J., Kavanagh J.J. (2011). Recurrence patterns and prognosis of endometrial stromal sarcoma and the potential of tyrosine kinase-inhibiting therapy. Gynecol. Oncol..

[75] Akahira J., Tokunaga H., Toyoshima M., Takano T., Nagase S., Yoshinaga K., Tase T., Wada Y., Ito K., Niikura H. (2006). Prognoses and prognostic factors of carcinosarcoma, endometrial stromal sarcoma and uterine leiomyosarcoma: A comparison with uterine endometrial adenocarcinoma. Oncology.

[76] Borella F., Bertero L., Cassoni P., Piovano E., Gallio N., Preti M., Cosma S., Ferraioli D., Pace L., Mariani L. (2022). Low-Grade Uterine Endometrial Stromal Sarcoma: Prognostic Analysis of Clinico-Pathological Characteristics, Surgical Management, and Adjuvant Treatments. Experience From Two Referral Centers. Front. Oncol..

[77] Scher D., Nghiem W., Aziz S., Rahbar R., Banks W., Venbrux A., Sarin S. (2015). Endometrial Stromal Sarcoma Metastatic from the Uterus to the Inferior Vena Cava and Right Atrium. Tex Heart Inst. J..

[78] Nam J.H. (2011). Surgical treatment of uterine sarcoma. Best Pract. Res. Clin. Obstet. Gynaecol..

[79] Harter P., Sehouli J., Reuss A., Baumann K., Hanker L., Kimmig R., Schröder W., Burges A., Gropp-Meier M., Kurzeder C. (2016). Phase II Study Evaluating PegLiposomal Doxorubicin and Carboplatin Combination Chemotherapy in Gynecologic Sarcomas and Mixed Epithelial-Mesenchymal Tumors A Phase II Protocol of the Arbeitsgemeinschaft Gynaekologische Onkologie Study Group (AGO-GYN 7). Int. J. Gynecol. Cancer.

[80] Kalender M.E., Sevinc A., Yilmaz M., Ozsarac C., Camci C. (2009). Detection of complete response to imatinib mesylate (Glivec/Gleevec) with 18F-FDG PET/CT for low-grade endometrial stromal sarcoma. Cancer Chemother. Pharmacol..

